# The gut-brain axis: is intestinal inflammation a silent driver of Parkinson’s disease pathogenesis?

**DOI:** 10.1038/s41531-016-0002-0

**Published:** 2017-01-11

**Authors:** Madelyn C. Houser, Malú G. Tansey

**Affiliations:** Department of Physiology, Emory University School of Medicine, Atlanta, GA USA

## Abstract

The state of the intestinal environment can have profound effects on the activity of the central nervous system through the physiological contributions of the microbiota, regulation of intestinal barrier function, and altered activity of peripheral neurons. The common language employed for much of the gut-brain communication is the modulation of immune activity. Chronic proinflammatory immune activity is increasingly being recognized as a fundamental element of neurodegenerative disorders, and in Parkinson’s disease, inflammation in the intestine appears particularly relevant in pathogenesis. We review the evidence that intestinal dysfunction is present in Parkinson’s disease and that it may reflect the earliest manifestations of Parkinson’s disease pathology, and we link these findings to dysregulated immune activity. Based on this, we present a model for Parkinson’s disease pathogenesis in which the disorder originates in the intestine and progresses with inflammation as its underlying mechanism. More in-depth investigations into the physiological mechanisms underlying peripheral pre-motor symptoms in Parkinson’s disease are expected to lead to the development of novel diagnostic and therapeutic measures that can slow or limit progression of the disease to more advanced stages involving debilitating motor and cognitive symptoms.

## Introduction

There is growing awareness within the scientific and medical communities of the strong connection between the status of the intestinal environment and the function of the central nervous system (CNS). This so-called “gut-brain axis” incorporates bidirectional communication between the central and enteric nervous and endocrine systems as well as regulation of immune responses in the gut and brain, and all aspects of this system appear to be heavily influenced by the activity of intestinal microbes.^1,2^ Much remains to be discovered regarding the content and consequences of the rich dialogue maintained between the CNS and the gastrointestinal (GI) system. Here, we focus on the potential for intestinal health to impact the brain and review evidence supporting the possibility that chronic intestinal inflammation may contribute to the development of neurodegenerative conditions such as Parkinson’s disease (PD).

### Mechanisms of intestinal modulation of CNS activity

Numerous mechanisms mediate correspondence between the brain and the intestine. The most direct path is via the vagus nerve, which originates with the dorsal motor nucleus in the medulla and extends through the abdomen to the viscera. The vagus nerve provides the primary parasympathetic control of basic intestinal functions, with abundant innervation of the stomach, small intestine, and appendix that decreases proximal to distal, terminating before the distal colon.^3^ Stimuli in the intestine can trigger vagal afferent signaling, which is a critical component of neuroimmune inflammatory reflex circuits that contribute to tonic peripheral immune regulation.^4^ Evidence also suggests that the vagus nerve may act as a direct conduit by which material from the intestine can pass to the brain.^5,6^

Increasingly, the vibrant microbial community that occupies the intestine is also being identified as a key regulator of CNS activity. Changes in the composition of intestinal bacterial populations have been associated with a wide array of conditions including neurological and neurodevelopmental disorders such as multiple sclerosis,^7^ autism, depression, schizophrenia and PD,^8^ and studies are beginning to explore some of the mechanisms that contribute to the powerful influence of the microbiota. Intestinal bacteria may exert direct effects on host processes through the production of signaling molecules that interact with the host nervous system, including hormones and neurotransmitters such as monoamines and GABA.^9,10^ It has been shown that shifts in intestinal microbiota composition can alter the levels of some of these molecules along with levels of growth factors and signaling proteins in the brain,^9^ creating the potential for significant functional alterations. The microbiome also plays a significant role in controlling the release of a variety of gut peptides such as leptin and neuropeptide Y from enteroendocrine cells. Many of these molecules can act on the host nervous system and in fact play a key role in regulating circadian rhythms, anxiety levels, and behavior.^10,11^ Gut bacteria are responsible for the conversion of primary bile acids produced by the liver to secondary bile acids, which are more readily absorbed through the intestinal epithelium. These bile acids can act as potent signaling molecules and regulate a variety of processes related to both the nervous and immune systems.^12^ Intestinal microbes are also the primary source of short-chain fatty acids (SCFAs). These molecules are known to significantly impact the gut environment and host metabolism and to exhibit potent anti-oxidant and anti-inflammatory properties.^2^ In rats, SCFAs such as butyrate have been linked to increased colonic motility.^13^ The presence of SCFA-producing bacteria in the intestine has even been shown to strengthen the blood-brain barrier (BBB) by promoting increased expression and organization of BBB tight junction proteins.^14^ Metabolites from intestinal microbes such as those described here have also been reported to alter host gene expression in the brain, providing additional avenues for the microbiota to influence the activity of the CNS.^9^ Interestingly, many, though not all, of the microbe-derived effects on the brain appear to be mediated through the vagus nerve.^15^

The activities of intestinal microbes are inextricably linked to the status of the intestinal immune system. Under normal, healthy conditions, mucus and a tight barrier of epithelial cells confine most microbes to the intestinal lumen or the epithelial surface. Here, they stimulate homeostatic immune responses which predominantly promote tolerance of commensal microbes and the maintenance of barrier integrity^14^ but do not cause significant inflammation, allowing microbes to persist in the intestine and execute their symbiotic functions.^16^ The introduction of inflammatory triggers can upset this delicate relationship, however. Damage to the intestinal tissue, the introduction of aggressive pathogens, or exposure to substances that provoke strong immune reactions can increase the inflammatory quality of the intestinal environment. In turn, enteric inflammation can induce a number of effects that ultimately alter CNS function.

Immune cells are capable of engaging in direct communication with neurons.^4^ The extent of the functional impact of neuro-immune synapses is not known, but it is clear that activated immune cells can modulate neuronal activity via the release of neurotransmitters and cytokines.^4^ Local effects of inflammation on enteric neurons stimulate CNS responses via the vagus nerve.^4,17^ Proinflammatory cytokines and activated immune cells in the circulation can also access the brain, particularly when the BBB is compromised, as it frequently is in aged individuals or in the context of neurodegenerative disease.^18–21^ Systemic inflammation can directly mediate BBB permeability. Extensive evidence has been reported linking molecules associated with inflammatory conditions, including cytokines, reactive oxygen species, matrix metalloproteases, and mediators of angiogenesis, with BBB disruption.^22^ Additionally, a positive feedback loop involving the traditionally proinflammatory cytokine IL-6 in conjunction with neuroimmune reflex circuits has been implicated in activating BBB “gateways” through which peripheral T cells gain access to the CNS.^20^ Breaches in the BBB can significantly alter immune responses to CNS antigens^23^ and compromise CNS protection against potentially harmful substances.

Perhaps the most well-characterized effects of intestinal inflammation on the CNS involve hyper-reactivity of the hypothalamic-pituitary-adrenal axis and imbalances in serotonergic activity.^24,25^ These changes have been associated with the manifestation of “sickness behavior” as well as anxiety and depression,^26,27^ and these psychological conditions are frequently observed as comorbidities in individuals with diseases characterized by persistent intestinal inflammation, such as irritable bowel syndrome (IBS) and inflammatory bowel disease (IBD).^25,28^ These and other systemic effects of intestinal inflammation are almost certainly mediated by a host of immune factors, but at present, the cytokines interleukin-1β (IL-1β), interleukin-6 (IL-6), and tumor necrosis factor (TNF) have been most frequently implicated.^26,27,29^ Studies in rodents as well as clinical trials in humans have demonstrated mitigation of these CNS changes induced by intestinal inflammation with specific inhibitors or antagonists of these cytokines,^26,30^ with cyclooxygenase inhibitors,^31^ and with disruption of vagal signaling.^29^

The systemic effects of intestinal inflammation may be further augmented by increases in intestinal permeability. Acute tissue injury may be incurred in a severe infection with an intestinal pathogen or in some forms of colitis, and this can cause temporary but substantial defects in the intestinal epithelial barrier. Low-grade inflammation normally induces more selective increases in paracellular permeability through regulation of tight junctions.^32^ Intestinal microbes regulate expression of barrier-promoting tight junction proteins,^14^ and many proinflammatory cytokines secreted by activated immune cells—including TNF, IL-1β, and IL-6—act on tight junctions to increase barrier permeability^33,34^ in order to facilitate recruitment of additional immune cells and components from circulation to sites of inflammatory insult. Weakening of the intestinal barrier allows broader engagement of the immune system, but it also compromises the containment of gut contents, particularly microbial products, which can leak from the intestine into the peritoneal cavity and into the circulation, eliciting systemic proinflammatory immune responses.^14^ Typically, if the source of the immune challenge is rapidly cleared, proinflammatory responses terminate, and gut barrier function is restored. Unique features of the intestine, however, render it particularly susceptible to the development of persistent inflammation and barrier dysfunction.

With roughly 100 trillion bacteria in the intestine along with abundant fungi and viruses, the intestinal immune system is constantly exposed to microbial antigens which may serve as stimuli that prolong inflammatory responses. If host immune tolerance of the microbiota is sufficiently disrupted, chronic disorders characterized by abnormal intestinal inflammation and permeability such as IBS or IBD may develop. Even if chronic disease does not manifest, low levels of intestinal inflammation such as those associated with obesity can significantly impact the microbiome, reducing diversity and altering bacterial population composition.^35^ Inflammation seems to promote the selective survival of more aggressive microbes that have mechanisms for subverting or tolerating proinflammatory host immune responses—qualities characteristic of pathogens.^35^ Thus, under inflammatory conditions, intestinal bacteria typically exhibit more pathogenic and less commensal activity, further exacerbating inflammation and increasing the likelihood of persistent immune responses in the intestine.

Sustained permeability of the intestinal barrier can have deleterious effects on numerous body systems. Many microbial components such as lipopolysaccharide (LPS) that enter the circulation with increased intestinal permeability are highly immunogenic and trigger systemic inflammatory responses. These responses, in turn, can promote further degradation of gut barrier function.^36^ The so-called “leaky gut syndrome” has been proposed as a contributing factor in a host of diseases. These include clearly GI-associated conditions like IBS, IBD, metabolic syndrome, and diabetes, but intestinal permeability is also being implicated in conditions of the CNS including autism, schizophrenia,^37^ multiple sclerosis,^38^ depression, anxiety, and post-traumatic stress disorder.^39^ Increases in systemic LPS can compromise both passive^40^ and active^41^ BBB mechanisms, rendering the CNS vulnerable to neurotoxic substances and activated immune cells from the periphery. Immune responses to circulating microbial antigens induce increases in proinflammatory cytokines in the periphery but also robust and persistent increases in the brain.^42^ This is likely facilitated at least in part by activation of microglia, CNS-resident immune cells.^42^ Proinflammatory responses in the brain can alter CNS function and behavior as previously described, and corrections in psychological and behavioral abnormalities accompanying resolution of inflammation and restoration of intestinal barrier function have been documented.^43^ CNS immune responses can have serious and enduring consequences, however, particularly if inflammation becomes chronic. Proinflammatory cytokines and oxidative stress have been causally linked to neuron death, and neuroinflammation is now considered a key factor in numerous neurodegenerative diseases.^44^

Many of these diseases are associated with advanced age, and as intestinal inflammation and forms of intestinal permeability have been documented to increase with age,^45^ immune mediation of gut-brain interactions may be particularly relevant in the pathology of neurodegenerative diseases of aging. One condition which has yielded some of the most substantial evidence of GI involvement is PD.

### Clinical features of PD

PD is diagnosed on the basis of classic motor symptoms that are caused primarily by the loss of striatal dopamine resulting from degeneration and death of dopaminergic neurons in the midbrain. These symptoms are typically treated with dopamine-replacement therapy, but no currently available treatment slows the progression of PD-related neurodegeneration. The precise causes of neurodegeneration in this disease have not been definitively established; however, abundant evidence has accumulated demonstrating the presence of neuroinflammation in PD patients, and glial cell activation, proinflammatory signaling molecules, and oxidative stress are now considered to be key mechanisms that contribute to neurodegeneration in PD.^46^ Another highly relevant factor in the pathogenesis of PD is the protein alpha-synuclein (αSYN). This molecule is present in numerous cell types throughout the body with high expression in presynaptic terminals of neurons, where it is thought to play a role in regulating vesicular release.^47^ New studies continue to revise our understanding of αSYN’s substantial conformational plasticity in normal physiology,^48^ but it is clear that under certain circumstances, this protein adopts a β-sheet structure, loses membrane-binding capacity, and aggregates. This leads to the histological hallmark of PD—Lewy neurites and Lewy bodies composed of fibrillar, phosphorylated, ubiquitinated αSYN.^49^ These aggregations are found upon autopsy in the brains of individuals with PD, Lewy body dementia, and multiple system atrophy, and less reliably in other neurodegenerative disorders.^49^ It remains unclear whether Lewy bodies are themselves neurotoxic or whether they form as a protective response to sequester toxic aggregated proteins from disrupting cellular organelles. The fact remains, however, that individuals with mutations in or over-expression of the αSYN gene *SNCA* usually develop early-onset, rapidly-progressing PD.^50^ The point mutations in *SNCA* associated with this phenotype stabilize β-sheet conformations, promoting aggregation with gain-of-function effects,^51^ and over-expression of αSYN has been shown to be sufficient to induce aggregation and neurodegeneration of dopaminergic neurons^52^ and is in fact the basis for several animal models of PD-like pathology. Given the abundance of evidence on the subject, it is quite likely that αSYN contributes either directly or indirectly to the pathogenesis of PD.

In addition to the well-established motor deficits, PD is also frequently characterized by an assortment of non-motor symptoms (NMS). One study determined that 98.6 % of PD patients report at least one NMS, and on average eight NMS were identified per person.^53^ The most common of these symptoms include hyposmia, constipation, anxiety, rapid eye movement sleep behavior disorder, depression, excessive daytime sleepiness, impaired reaction time, and impaired executive function.^54^ Some NMS in PD may be additional consequences of deficiencies in CNS dopaminergic activity or side effects of dopamine replacement therapy, but other NMS cannot be accounted for in this way, and as such may provide insight into underlying pathological mechanisms in PD. Furthermore, NMS are often present in pre-clinical stages and have been observed with greater frequency in individuals who later develop PD compared to those who are not diagnosed with this condition,^54^ suggesting that NMS may be manifestations of the earliest stages of PD, before dopaminergic neurons in the midbrain are affected. Recognizing pre-motor elements of this disease and defining mechanisms that regulate them may offer the potential for earlier diagnosis and more timely therapeutic intervention that could delay or even prevent the development of the progressive motor symptoms of PD.

### Intestinal involvement in PD

Recent evidence suggests that intestinal dysfunction is a non-motor symptom consistently associated with PD that may precede motor symptoms by decades. Constipation is the most common GI complaint^55^ and the second most common NMS behind hyposmia in PD.^54^ Studies report constipation in 20-80 % of PD patients,^56,57^ and a meta-analysis places the incidence at 50 %.^54^ Constipation in PD is likely due in large part to prolonged intestinal transit time, which has been reported to affect both the small intestine^58^ and colon.^59^ Intestinal motility is largely controlled by the enteric nervous system (ENS),^60^ but there is presently no consensus on whether PD-associated constipation occurs as a result of neurodegeneration within the ENS, the CNS, both, or as a consequence of another process entirely. It is clear, however, that constipation can manifest as a pre-motor symptom years before CNS degeneration prompts a diagnosis of PD. One study reported that middle-aged men who had less than one bowel movement per day had over four-fold increased risk for PD diagnosis over the next 24 years compared to men with regular bowel movements.^61^ Another study found that constipated men (three or fewer bowel movements per week) were five times more likely and constipated women three times more likely to be diagnosed with PD within 6 years compared to individuals who were not constipated.^62^ Meta-analyses suggest that constipation is more than twice as common in people who develop PD compared to those who do not,^54^ and that constipated individuals are twice as likely to develop PD within 10 years of their evaluation.^63^ The duration of time over which constipation is predictive of PD development is remarkable, but both prospective and retrospective studies have found that constipation becomes apparent at an average of 15.6–24 years before PD is diagnosed,^57,61,64,65^ making it one of the earliest indicators of pathological processes that ultimately lead to PD.

Another intestinal feature of PD that has been widely reported is the presence of enteric abnormalities in αSYN. This protein is expressed as a normal component of the ENS, and it can be detected in intestinal tissue in a large percentage of neurologically intact humans,^66–68^ with levels potentially increasing with age.^68^ Numerous studies indicate, however, that αSYN is detected more frequently and at higher levels in the intestines of PD patients than in age-matched healthy controls.^66,69–73^ This is significant, as over-expression of αSYN is known to produce αSYN aggregation in both the intestines and brains of mice and humans.^50,74,75^ Instances of phosphorylated and aggregated αSYN have been observed in the esophagus, stomach, small intestine, colon, and rectum of PD patients, but it has been suggested that they occur in a proximal to distal gradient, with the lowest frequency in the rectum.^76,77^ Studies that have evaluated enteric αSYN in PD patients have reported detection of phosphorylated αSYN in 61.580 % of PD samples and Lewy bodies/Lewy neurites in 72.4–100 % of PD samples, compared to detection in only 0–33 % of healthy controls.^77–81^ This would suggest that intestinal synucleinopathy is a relatively sensitive and specific indicator of PD pathology. Furthermore, additional studies have reported distinctive αSYN immunoreactivity in intestinal biopsies taken from clinically normal individuals who would later develop PD,^69–71^ indicating that abnormal enteric αSYN is present before CNS neurodegeneration has advanced sufficiently to produce motor symptoms.

These conclusions regarding the distinctive features of intestinal αSYN in PD are not universally accepted. Some researchers argue that phosphorylated αSYN and Lewy bodies in the intestine are not specific features of PD as they have been identified in the intestine of patients with other disorders such as achalasia,^82^ Lewy body dementia, incidental Lewy body disease, and Alzheimer’s with Lewy bodies.^77^ It is worth noting, however, that these disorders share many pathological features with PD, and it has been proposed that at least some of these neurological conditions may actually represent early, pre-motor stages of PD.^83,84^ This concept may be supported by observations that the extent of intestinal synucleinopathy in these conditions is not as advanced as that observed in the GI system of PD patients.^77^

Variability in the findings regarding enteric αSYN in different studies may be attributable to numerous factors, including qualifications for subject inclusion or exclusion, the age of the subjects, stage of PD, the tissue type and the particular mucosal plexus examined, preparation of the samples, the type of assay employed, the antibody or stains used, and the definitions applied to categorize samples as “positive” or “negative.” Furthermore, it is not clear whether sex differences in intestinal synucleinopathy exist or whether the manifestations of pathology may differ in inherited and idiopathic PD, as these distinctions are rarely reported in the current literature. Reports on this topic also focus exclusively on αSYN in enteric neurons, though it is also expressed and may have significant PD-associated effects in other cell types, such as in immune cells, or in other tissues besides the gut and the brain. Finally, it may be valuable to consider not just the presence or absence of enteric αSYN and synucleinopathy, but also the aggregated αSYN load. Particularly if PD represents an extreme on a spectrum of αSYN abnormalities, then levels of expression, degree of post-translational modification, proportions of affected cells, and abundance of aggregates may provide greater insight into the role of this protein in disease pathogenesis than do the binary data currently reported in the literature. Before enteric synucleinopathy could be considered for use as an early risk indicator for PD, more extensive studies must be done, and optimal techniques for tissue collection and processing must be determined and standardized.

Another intestinal component of PD that is receiving increasing attention is alterations in barrier function. A number of reports now indicate that PD patients have increased intestinal permeability compared to healthy controls.^39,73,81,85,86^ The results obtained from several of these studies specifically indicate defects in intestinal tight junctions without gross mucosal damage, including reductions in levels of barrier-promoting proteins and disruptions of tight junction networks,^81,85,86^ a phenotype consistent with low-grade inflammation.^32^ Defects may also be present in other intestinal barriers that limit direct microbial interaction with tissue, as instances of *Escherichia coli* penetration into the intestinal mucosa were more frequent in PD patients than controls and correlated, as might be expected, with increases in intestinal permeability and with oxidative stress.^73^ Interestingly, levels of enteric αSyn were also found to correlate positively with gut permeability.^73^ No reports have yet been published that assess intestinal barrier function in asymptomatic individuals who later develop PD, so the extent to which increased permeability is a pre-motor symptom of PD has not been firmly established. Increased intestinal permeability was detected in newly-diagnosed PD patients, though, so it can be concluded that it is present at least from the earliest clinical stages of the disease.^73^

In recent years, the relationships between intestinal microbes and PD pathology have become subjects of active investigation. A greater incidence of small intestinal bacterial overgrowth (SIBO) consisting of increased bacterial density and dysbiosis in the small bowel has been reported in PD patients compared to unaffected controls.^87–90^ The prevalence of SIBO in PD patients in these studies ranged from 25–54.5 % compared to 8.33–20 % in unaffected controls. One study reports greater frequency of SIBO in recently-diagnosed PD patients,^89^ while another found increased frequency with advanced disease,^88^ so there is no consensus on when this symptom develops in the course of the disease. As SIBO can be caused by the impairment of enteric-migrating motor complexes that reduce intestinal motility,^91^ it may be that this condition emerges when PD-associated neuropathology in the intestine has advanced sufficiently to increase small intestine transit time. This is supported by the fact that SIBO is also common in diabetes mellitus, another condition associated with impaired GI motility.^92^ The presence of SIBO has been associated with greater motor impairment in PD^89^ and with fluctuating responses to levodopa, and antibiotic treatment has been shown to temporarily improve these symptoms.^87^

Only very recently have more detailed assessments of the intestinal microbiota in PD been initiated. Four studies have been published that characterized the composition of fecal or intestinal bacteria populations in PD, and from these, meaningful patterns are beginning to emerge. When comparing the microbiota in feces or colon biopsies, more differences were found between PD patients and controls in fecal rather than tissue-adherent bacteria.^93^ Some of the reported differences included decreased abundances of fecal *Bacteroidetes*, *Lactobacillaceae, Faecalibacterium prausnitzii, Enterococcaceae,* ^94^ *Prevotella*,^94,95^ and *Clostridium spp* ^85,93^ in PD, increased abundances of *Bifidobacterium* and *Lactobacillus spp*,^85^ and increased *Enterobacteriaceae* in PD patients^94^ with potential specificity for those exhibiting postural instability and gait difficulty as prominent symptoms.^95^ One study reported that relative abundances of four bacterial families (*Prevotellaceae*, *Lactobacillaceae*, *Bradyrhizobiaceae*, and *Clostridiales Incertae Sedis IV*) could be used to identify PD cases with over 90 % specificity but only 47.2 % sensitivity, suggesting that this microbial signature may be representative of at least a subset of PD patients.^95^ Metagenomics analysis indicates that functional effects of these microbiota changes in PD include a significant reduction in normal metabolic activities and a significant increase in resources devoted to synthesis of cell wall component LPS and to type III bacterial secretion systems,^93^ which are often involved in pathogenic interactions with host cells. In short, the fecal microbiota composition in PD patients appears deficient in microbes that mediate mutualistic anti-inflammatory and metabolic activities and enriched in pathobionts that stimulate inflammation and may induce damage to host tissue.

As noted in these studies, organisms in the *Prevotella* and *Clostridiales* taxa that are reportedly less abundant in PD patients are prominent producers of SCFAs, such as butyrate as well as folate and thiamine, all of which have been associated with amelioration of PD pathology.^95^ In support of this, a recent study confirmed reduced concentrations of SCFAs butyrate, acetate, and propionate in feces of PD patients compared to controls that exceed the reductions associated with normal aging.^94^ Though members of the *Enterobacteriaceae* family that is more abundant in at least a subset of PD patients can be a component of the normal microbiota, this taxon has also been clearly linked to inflammation and disease. *Enterobacteriaceae* levels in the intestine correlate strongly with levels of inflammation indicators.^96^ The relative abundance of *Enterobacteriaceae* surges when microbial diversity is reduced, as it often is in inflammatory or dysbiotic conditions. Furthermore, *Enterobacteriaceae* levels in PD were positively correlated with the severity of certain motor symptoms,^95^ suggesting that these microbes or the inflammation they promote contribute directly to PD pathology.

At this time, it is not clear whether the observed changes in microbiota in PD patients are an initial occurrence that contributes to the development of neurological dysfunction and degeneration, or if they emerge in response to PD-related pathology in the enteric and/or the CNSs that impairs peristaltic activity and induces inflammation. Both may be possible, as a recent study neatly demonstrated, finding that congenital intestinal immune dysregulation and prolonged intestinal transit time in a mouse model altered the composition of gut microbial populations and that those dysbiotic flora could then recapitulate intestinal dysfunction in wild type mice.^97^ In PD patients, levels of butyrate-producing *Lachnospiraceae* were negatively correlated with duration of PD,^93^ suggesting either that these beneficial microbes are lost over the course of the disease or, intriguingly, that greater abundances of certain taxa may delay the onset of clinical symptoms of PD. Genetic studies provide additional support for the connections between enteric microbes, immune responses, and disease. Many of the genetic variants linked to familial as well as sporadic PD impact inflammatory responses,^98^ and these may be especially relevant in the gut. There is an intriguing degree of overlap between genetic risk factors for Crohn’s disease (CD)—a chronic intestinal inflammatory reaction against the microbiota—and PD. Variants in the *LRRK2* gene are common in both CD and PD, and while the pathogenic mechanisms are not well-understood, like other PD genetic risk factors, LRRK2 is known to regulate inflammatory responses.^99^ Several *CARD15/NOD2* SNPs are associated with CD and contribute to disease risk, age of onset, and pathological manifestations, with dysregulated NFκB activity as a possible mechanism. At least one of these same variants is also over-represented in PD patients.^100^ A recent study suggests that the overlap between CD and PD risk loci extends beyond these known shared associations,^101^ so there may be even greater similarity in genetic predisposition to this intestinal inflammatory and this neurodegenerative disorder.

The temporal relationships among gut microbes, intestinal dysfunction, and the development of PD have also been debated with regard to lifestyle factors linked to PD risk. Specifically, both smoking and drinking coffee are associated with significantly reduced incidence of PD, and it has been proposed that protective effects of tobacco and coffee may be mediated at least in part by modulation of the microbiota.^92,102^ Some evidence now suggests that changes in certain bacterial taxa that are observed with smoking and coffee consumption resemble the microbiota differences reported in controls compared to PD cases.^102^ This raises the possibility that some states of the microbiome may be protective with regard to PD, but it is also possible that the smoking and coffee-associated microbial compositions reflect changes in the intestinal environment or function. Nicotine has well-established anti-inflammatory properties, and coffee increases gut motility,^102^ both of which may contribute to an intestinal environment that is resistant to PD-associated pathology. Alternatively, some have suggested that PD-related impairment of neurochemical reward systems may lead individuals with PD, even in the pre-motor stages, to consume less nicotine and caffeine,^92^ rendering associated intestinal differences an artifact of lifestyle activities. This explanation is not entirely satisfactory, however, as some PD-associated differences in bacterial populations persist even when factors such as smoking practices are accounted for.^95,102^ In spite of the many unanswered questions regarding the role of intestinal microbes in the development of PD, because of their known potential to impact the CNS, and because differences in their relative abundances have been associated with PD symptoms,^95^ they represent a potential target for intervention that could at least help mitigate symptom severity and at best, inhibit PD pathogenesis.

### Model of gut-originating, inflammation-driven PD pathogenesis

**Fig. 1:**
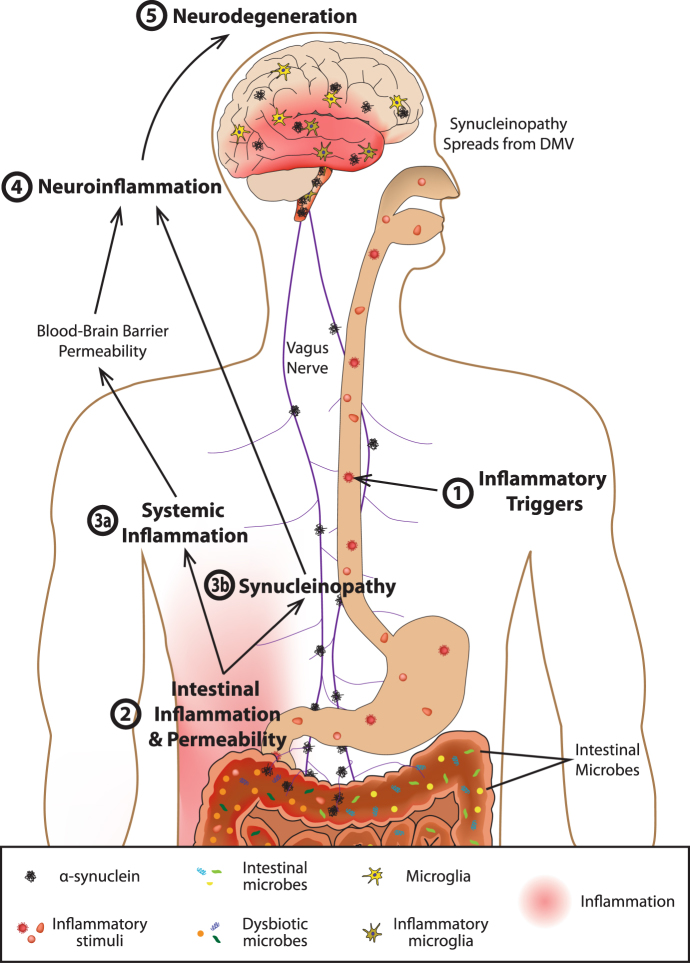
**Model of gut-originating, inflammation-driven PD pathogenesis.** In a susceptible individual, inflammatory triggers (1) initiate immune responses in the gut that deleteriously impact the microbiota, increase intestinal permeability, and induce increased expression and aggregation of αSYN (2). Synucleinopathy may be transmitted from the gut to the brain via the vagus nerve (3b), and chronic intestinal inflammation and permeability promote systemic inflammation, which, among other things, can increase blood-brain barrier permeability (3a). Intestinal inflammation, systemic inflammation, and synuclein pathology in the brain all promote neuroinflammation (4) which drives the neurodegeneration that characterizes PD (5)

We have described how CNS function is influenced by intestinal microbes and the molecules that they produce which together act to stimulate enteric immune activity and regulate gut permeability. Sustained inflammatory conditions in the intestine can promote systemic inflammation and neuroinflammation. PD is characterized by intestinal dysfunction that can start over two decades before the onset of motor symptoms, and the observed constipation, intestinal permeability, dysbiosis, and increased levels of potentially pathogenic forms of enteric αSYN are all consistent with conditions of GI inflammation.^32,35,103,104^ In fact, studies have demonstrated that PD patients do exhibit inflammation and oxidative stress in the gut.^73,105^ On the basis of this accumulated information, the following model of PD pathogenesis can be formulated (Fig. 1).

#### An initial inflammatory trigger is introduced

This could be a toxic substance (such as the pesticides or pollutants that have been associated with PD^1^) that induces damage and subsequent inflammation in the intestine. Many have proposed that an infection, which directly or indirectly affects the GI system could be the initiating factor; or perhaps it is more accurately the accumulated inflammatory burden of multiple infections, which has recently been linked to PD.^106^ Even IBS or IBD, which have been reported to increase risk for PD development,^107,108^ could serve as the catalyst for a proinflammatory intestinal immune response that could ultimately promote the development of PD.

#### Sustained, low-level inflammation develops

If the immune responses elicited by the trigger are not resolved promptly, they would be expected to contribute to deleterious shifts in microbiota composition and to intestinal permeability which would allow leakage of microbial products and inflammatory mediators from the intestine. These could prompt systemic immune responses which, among other consequences, could increase the permeability of the BBB. Systemic inflammation has, in fact, been commonly reported in PD patients,^109–112^ as has BBB dysfunction, particularly in late-stage disease.^21,113^

#### Synucleinopathy develops and exacerbates inflammation

Proinflammatory immune activity and conditions that elicit it have been shown to increase levels of αSYN in the gut and the brain.^104,114^ αSyn over-expression would then trigger its aggregation.^52^ Over-expressed and aggregated αSYN would in turn stimulate proinflammatory responses from immune cells, initiating a positive feedback loop that could promote the spread of aggregated αSYN to other tissues.^115,116^

#### αSYN pathology in the periphery can transfer to the brain

While prolonged systemic inflammation may on its own be sufficient to pathologically modify αSYN in the CNS, peripheral inflammation may also increase uptake of αSYN from circulation into the CNS by promoting disruption of the BBB.^117^ Furthermore, it was recently demonstrated that human αSYN introduced into the intestinal wall of rats could migrate up the vagus nerve to the dorsal motor nucleus in the brainstem.^6^ This translocation was mediated by microtubule-associated transport in neurons, and it was observed equally for monomeric, oligomeric, and fibrillar forms of αSYN.^6^ This proof-of-concept study suggests that changes induced in αSYN in the gut can directly affect the brain via the vagus nerve. In the brain, αSYN activates microglia^115^ which may already be primed by the ongoing GI and systemic immune responses. It has been shown that peripheral inflammation exacerbates inflammatory responses to αSYN in the CNS,^116^ increasing the likelihood and accelerating the timeline in which neuroinflammation produces neurodegeneration.

#### CNS pathology in PD begins in the dorsal motor nucleus of the vagus (DMV)

Numerous reports indicate that the DMV is consistently affected in PD patients,^60^ exhibiting αSYN inclusions^118^ and neurodegeneration.^119^ Heiko Braak and colleagues proposed a staging system for the progression of PD pathology through the brain with involvement of the DMV as the first stage.^120^ Questions have been raised regarding Braak’s staging scheme and the methodology of his study. Numerous publications have provided support, however, for much of Braak’s staging, and studies designed to refute his proposal have still found that a majority of PD cases (53–81.7 %) adhere to it fully, and that only a small fraction (7–8.3 %) do not show pathology in the DMV when it is present elsewhere in the brain.^121,122^ Thus, it would appear that the majority of PD patients exhibit a form of the disease that follows a specific pathological progression in which the DMV is prominently affected. It is possible that the small percentage of deviations from standard findings could result from differences in disease manifestations between inherited, possibly monogenic forms of PD and sporadic cases, as few studies report these categorizations.

#### PD pathology spreads throughout the brain

From the DMV, synucleinopathy, inflammation, and neuronal dysfunction would then propagate to other brain regions, ultimately reaching the substantia nigra, where dopaminergic neurons, which are particularly sensitive to inflammation,^115^ begin to degenerate. When sufficient depletion of striatal dopamine resulting from loss of these dopaminergic neurons has occurred, motor impairments begin to manifest, and clinical symptoms of PD have developed.

This is only one model of PD pathogenesis that may apply in only a subset of patients, but it has robust support in the literature, and its elements are becoming increasingly common proposals in the field of PD research.^93^ In addition to the collection of intestinal symptoms long associated with PD that suggest a gut origin for the disease, it was reported several years ago that in a mouse model with over-expression of mutant human αSYN, enteric abnormalities appeared before any CNS pathology.^123^ In another study, when rotenone, a pesticide commonly used to induce parkinsonian pathology in rodents was delivered into the stomach of mice, it was found to induce enteric neurons to release αSYN, which then propagated to other neurons by retrograde axonal transport,^124^ accompanied by local inflammation.^125^ αSYN accumulation and phosphorylation appeared sequentially in the ENS, the DMV, and then other brain regions in accordance with Braak’s proposed PD staging.^125^ This progression could be halted by resection of autonomic nerves.^124^ Accordingly, severance of the vagus nerve was recently found to be associated with reduced risk of PD in humans.^126^ The inflammatory aspects of PD are now well established, and the concept of immune activation originating in the gut can link numerous risk factors, pre-motor, non-motor, and motor symptoms into a cohesive hypothesis of PD pathogenesis that can explain much the observed disease presentation.

## Conclusions

Over the years, our understanding of PD has evolved from identification of an impairment of midbrain neurons to recognition of a multi-system disorder with central and peripheral, motor, non-motor, and pre-motor manifestations, many of which center in the GI tract. A preponderance of evidence now suggests that the intestines are not only affected in PD, but that this may be the site where pathology initiates decades before progressing from the enteric to the CNS. Furthermore, the connections between the nervous and immune systems and between inflammation and neurodegeneration have become far too substantial for modern PD research to ignore. Recognition of the complexity of PD offers new insight into its mechanisms. It is reasonable to speculate, for instance, that molecular regulators of intestinal, CNS, and systemic inflammation may play key roles in PD pathogenesis. The involvement of intestinal inflammation in PD presents opportunities for the development and application of novel diagnostics and earlier therapeutic interventions. This may include identification of peripheral biomarkers and clinical investigations with immunomodulatory drugs that, if administered early enough, could be effective by acting on peripheral sites of pathology without the need to cross the BBB. Targeting pre-motor intestinal stages of PD for therapeutic intervention may hold the potential to slow or halt progression of the disease to the CNS and would significantly increase and prolong quality of life for PD patients and their families.
